# A Case of Suppurating Epididymitis

**Published:** 1881-10

**Authors:** E. I. Brackett

**Affiliations:** Lewiston, Me.


EDITOR OF JOURNAL OF COMPARATIVE MEDICINE:

### VIII.—A CASE OF SUPPURATING EPIDIDYMITIS.

EDITOR OF JOURNAL OF COMPARATIVE MEDICINE:

I was recently called by telegram to Bangor, Me., to visit a diseased horse, and it proved to be a very singular case.

I found the horse with a running sore about five inches posterior to the scrotum. I think they told me it had been running about two years. I cast the horse and made an incision at the opening of sore, followed it from three to five inches, and found the testicle grown to the walls. I dissected it out, and found the epididymus externally in almost a normal condition, but filled with pus internally. I operated on him three weeks ago; he is now at work, and well healed.

E. I. BRACKETT, V.S.

LEWISTON, Me.

